# A Multidisciplinary approach to metastatic giant basal cell carcinoma—A case report

**DOI:** 10.1016/j.jpra.2024.03.003

**Published:** 2024-03-08

**Authors:** Leo E.T. Lehto, Marko T. Ristola, Veera Mäntylä, Susanna Pajula

**Affiliations:** a Turku University Hospital, Department of Plastic and General Surgery, Finland; b Satasairaala Hospital, Department of Surgery, Finland

**Keywords:** Case reports, Basal cell carcinoma, Neoadjuvant therapy, Vismodegib, Non-melanoma skin cancer, Metastatic

## Abstract

We present a case of a 49-year-old man with a giant basal cell carcinoma of the back, with metastases in the lungs, liver, mediastinum and both adrenal glands. Neoadjuvant vismodegib was administered, after which wide local resection of the tumour was performed. There have been no signs of local recurrence.

## Introduction

While basal cell carcinoma (BCC) is the most common type of cancer in the world, they are usually slow-growing, and the rate of metastasis is remarkably low at 0.0028–0.55 %.^1^, ^2^, ^3^ Giant Basal Cell Carcinomas (GBCC), defined as 5.0 cm or more in maximum dimension, are much more aggressive, and with tumours greater than 10 cm, the incidence of metastases and/or fatal outcome may be as high as 45 %.^3^^,^^4^ If the tumour is allowed to grow for several years, it may reach a substantial size, infiltrate deeper structures, and metastasize, especially in the case of infiltrative and morpheaform BCC.^5^, ^6^, ^7^, ^8^ The most common sites for metastases are lymph nodes, lung, and bone.^1^

A whole body CT has been recommended to detect metastases in GBCC.^3^^,^^9^ Patients with locally advanced BCC or metastatic disease, hedgehog pathway inhibitors vismodegib or sonidegib should be considered.^6^

Our case report supports the whole body CT scan, vismodegib as adjuvant therapy and wide local excision of the tumour. A sentinel node biopsy was unnecessary in our case.

## Case report

### Anamnesis

**Figure 1:**
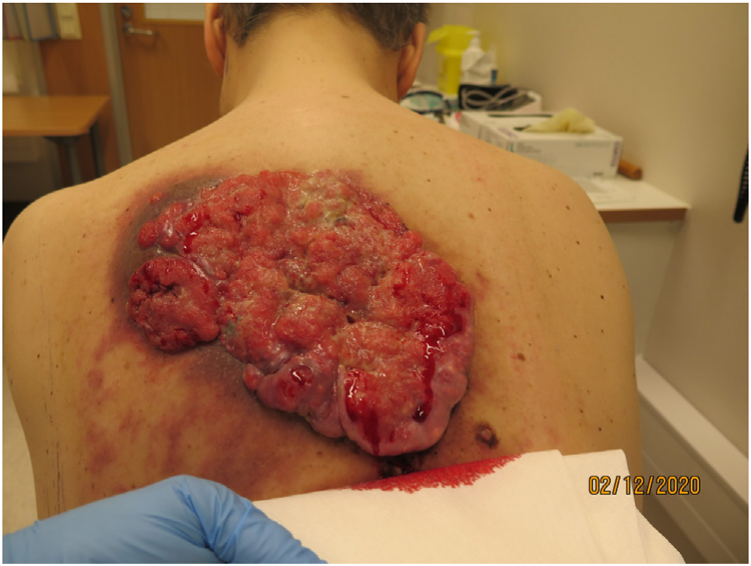
Preoperative photograph of the tumour.

A 49-year-old male with no previous medical history came to a Finnish central hospital emergency room in September 2020 due to dyspnea and angina pectoris. The patient reported having had a bleeding ulcer in his back for six years (Figure 1) which had resulted in microcytic anaemia. Hemoglobin level was 85 (134–167) g/l. In the physical examination a 20 × 20 cm tumour was found on his back that was strongly suspicious for malignancy.

### Diagnosis

The next day a whole body CT scan was performed and skin biopsies were taken. The CT scan showed enlarged lymph nodes in the mediastinum, and suspected metastases in the liver and both adrenal glands.

The skin biopsies showed BCC with infiltrative features. The suspected liver metastasis was not visible with ultrasound or MRI, and was also poorly visible in the CT scan and therefore biopsy was not able to be performed.

### Treatment

**Figure 2:**
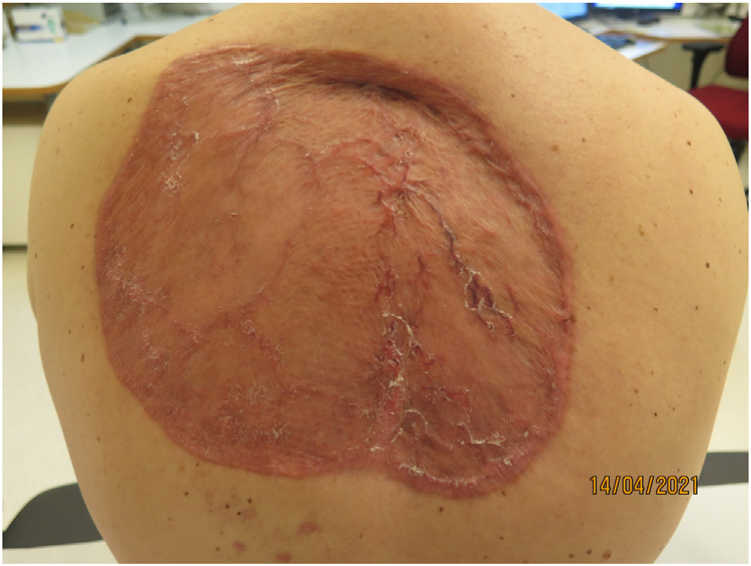
Postoperative result 3 months after surgery.

The University Hospital multidisciplinary skin cancer team was consulted and an oral treatment of Vismodegib 150 mg daily was started by the oncologist in December 2020. Surgical treatment was performed in January 2021. The primary tumour was excised with wide margins and negative frozen sections biopsies. The defect was reconstructed with meshed split thickness skin grafts (STSG) from the patient's thighs. The cosmetic and functional outcome of the surgery at 3 months was acceptable (Figure 2).

### Follow-up

Later histopathology confirmed nodular BCC with infiltrative features. Histopathological margins were 13,5 mm to the side and 10,5 mm to the depth. The patient had follow-up visits with an oncologist due to Vismodegib treatment which was continued until March 2022. There were no adverse effect of Vismodegib.

A CT scan in December 2022 showed that lesions in the liver were no longer visible and suspected metastases in the adrenal glands were the same size as in the previous follow-up CT. However, originally a 4 mm apical nodus laterally in the right lung was now 5.5 mm in diameter and suspicious for metastasis. The tumour was resected thoracoscopically for histopathological diagnosis in January 2023. Histopathology was inconclusive: a metastatic lesion of the GBCC could not be ruled out. A follow-up CT scan performed in September 2023 showed no progression.

## Discussion

In this case report we presented a 49-year-old man who had developed a giant basal cell tumour on his back over a period of six years, causing anaemia and it had metastasized. The possibility of a neglected malignant skin tumour must be kept in mind when treating patients with anaemia even in emergency care setting.

Mohs micrographic surgery (MMS) is also used in BCC surgery. In our case, due to size of the tumour and location in the back radical excision had performed. STSG reconstruction was chosen because of its ability to cover large defects.

A whole body CT is recommended in GBCC's and was useful tool to assess metastases also in our case.^3^ Our patient was already diagnosed with distant metastases preoperatively and was started on vismodegib treatment. Hence, sentinel node biopsy was not performed.

While our patient did not have a complete response to vismodegib, some of the metastases vanished altogether and some decreased in size. A European guideline supports the use of Vismodegib.^10^

## Conclusion

Even though most BCC's are not aggressive they may grow into aggressive tumours. Early detection of malignant skin tumors is crucial for the patient's treatment.

## Informed consent

Before submitting this paper, the group acquired informed consent from the patient.

## Declaration of competing interest

None.
